# Personalized (N-of-1) Trials for Patient-Centered Treatments of Multimorbidity

**DOI:** 10.1162/99608f92.d99e6ff5

**Published:** 2022-09-08

**Authors:** Jerry M. Suls, Catherine Alfano, Christina Yap

**Affiliations:** 1 Institute of Health System Science, Feinstein Institutes for Medical Research, Northwell Health; Manhasset, NY, USA; 2 Northwell Health Cancer Institute; Manhasset, NY, USA; 3 Clinical Trials and Statistics Unit, The Institute of Cancer Research; London, UK

**Keywords:** cancer, coronavirus disease 2019 (COVID-19), multimorbidity, personalized (N-of-1) trials, randomized controlled trials (RCTs), comorbidity

## Abstract

Treatment of patients who suffer from concurrent health conditions is not well served by (1) evidence-based clinical guidelines that mainly specify treatment of single conditions and (2) conventional randomized controlled trials (RCTs) that identify treatments as safe and effective on *average*. Clinical decision-making based on the average patient effect may be inappropriate for treatment of those with multimorbidity who experience burdens and obstacles that may be unique to their personal situation. We describe how the personalized (N-of-1) trials can be integrated with an automatic platform and virtual/remote technologies to improve patient-centered care for those living with multimorbidity. To illustrate, we present a hypothetical clinical scenario—survivors of both coronavirus disease 2019 (COVID-19) and cancer who chronically suffer from sleeplessness and fatigue. Then, we will describe how the four standard phases of conventional RCT development can be modified for personalized trials and applied to the multimorbidity clinical scenario, outline how personalized trials can be adapted and extended to compare the benefits of personalized trials versus between-subject trial design, and explain how personalized trials can address special problems associated with multimorbidity for which conventional trials are poorly suited.

## 1. Introduction

Clinical medicine lacks a fully satisfactory way to deliver personalized care that efficiently addresses the needs of patients with concurrent multiple health conditions. In this article, we describe how the personalized (N-of-1) trial can improve these patients’ care and personal empowerment. An estimated 145 million Americans had two or more concurrent health conditions in 2009, and the number is expected to rise to 171 million by 2030 (Goodman et al., 2013; King et al., 2018; McPhail, 2016). Evidence shows that multimorbidity increases illness burden, risk of premature death, and health care costs (e.g., Suls et al., 2019; Wolff et al., 2002), though clinical practice guidelines focus on the management of single diseases (e.g., Fortin et al., 2006). This stems from reliance on evidence from randomized between-subject controlled trials that enroll relatively homogeneous samples to reduce the confounding role of other medical conditions (e.g., Jadad et al., 2011; Kronish et al., 2018).

Lacking guidelines for patients with concurrent health conditions, medical practitioners rely upon ad hoc combinations of therapies formulated for each component disease. An untested combination of treatments, however, can increase medical problems, side effects, patient burden, polypharmacy, and inefficiencies in care delivery (Boyd & Kent, 2014).

A more fundamental problem, even if conventional randomized controlled trials (RCTs) recruited more participants with multiple health conditions, is the widespread assumption that between-subject treatment change is roughly equivalent to within-subject treatment change (Davidson et al., 2014). However, between-subject change indicates only that a treatment is safe and effective on *average.* A treatment, even one associated with a statistically significant overall benefit, may have an uneven mix of risks and benefits to individual patients (i.e., heterogeneity of treatment effect [HTE]) (Greenfield et al., 2007; Kent et al., 2018; Kravitz et al., 2004); treatments may incur substantial benefits, modest or no effect, and/or harms. Stratifying trial populations by primary outcome risk or treatment-related harms (Kent et al., 2018) can offer more precision, but identify, at best, only subgroups of patients.

This imprecision impedes contemporary medicine’s mission to provide patient-centered care with good outcomes that are “meaningful and valuable to the *individual patient*” (emphasis added) (Epstein & Street, 2011, p. 100; Guyatt et al., 2004). Such care may be critically important for patients with multiple concurrent health conditions and who experience burdens and obstacles that may be unique to their personal situation. As Boyd and Kent (2014) note, “multimorbidity poses a fundamental challenge to the reductionist paradigm of [evidence-based medicine]. . . New paradigms may be needed to address these challenges” (p. 553). As a solution to improve patient-centered care, we propose personalized trials, a classic research paradigm, renovated by advances in technology, statistics, and computing (Davidson et al., 2014; Kronish et al., 2017; Lillie et al., 2011).

## 2. Personalized (N-of-1) Trials

In a personalized trial, the effect of one treatment is compared with one or more other treatments or a placebo condition, all experienced by a single person, and the within-individual differences are calculated. These designs are essentially multiple crossover trials conducted to identify the optimal treatment for a single patient (i.e., rather than the greater population) (Guyatt et al., 1986; Sedgwick, 2012). The patient is randomized to receive one or more reversible interventions with a control condition; in a random order; and, in some cases, a washout period (i.e., an interval during which subjects receive no treatment so the effects of a previous treatment are eliminated). Since each patient serves as their own control, these crossover trials reduce confounding by between-subject covariates.

The within-subject, single-patient design can offer direct, objective evidence about the usefulness of a particular treatment for a specific patient. The experience and individualized feedback afforded to patients can also empower them to actively participate in selecting their treatment options, primary elements of patient-centered care and patient engagement (Nikles et al., 2005; Shamseer et al., 2016).

Personalized experimental designs were first used successfully in the early days of behavioral science. Previous isolated attempts at personalized clinical trials in medicine have been for single symptoms with a single protocol, and they were run for a few dozen patients. When one successful use case was found, the clinician had to continue to be the sole provider and manually conduct most aspects of that trial for all future patients. Typically, personalized trials require patients to receive instructions and monitoring on a frequent and periodic basis, which is time and labor intensive. This was not sustainable, and all previous attempts at incorporating any personalized approach into clinical medicine have not been maintained—including one that Gordon Guyatt started at McMaster University in the 1980s, one that Eric Larson championed at the University of Washington in the 1990s, and one at University of Alberta in the early 2000s (Mirza et al., 2007). The root cause of these implementation failures was the lack of any automated, sustainable, user-friendly technology platform that renders the conduct of such a trial facile. Advances in computing and technology, however, make such platforms possible. Herein, we illustrate how personalized trial methodology, integrated with virtual and remote technologies, can inform care of patients with multimorbidity using a hypothetical clinical scenario as an example. We outline how the four standard phases of conventional RCT development are modified for personalized trials—using the clinical scenario as an illustration—how personalized trials can be adapted and extended to compare the benefits of personalized trials versus between-subject trial design, and explain how personalized trials can address special problems associated with multimorbidity for which conventional trials are poorly suited.

## 3. A Clinical Scenario: Survivors of COVID-19 and Cancer

For our clinical scenario, we purposely choose a contemporary public health challenge faced by patients and the health care professionals who serve them. In recent decades, improvements in treatment have increased survival among persons diagnosed with cancer (American Cancer Society, 2020) and rapid medical advances in the past 24 months have done the same for patients infected with coronavirus disease 2019 (COVID-19) (e.g., Oliveira et al., 2021). This success has led to an increased prevalence of patients who have survived both cancer and COVID-19. Survival does not, however, mean these patients do not experience new or residual symptoms months and perhaps years post-hospitalization. Fatigue and sleep disruption are frequently reported as very distressing residual symptoms for those persons living with cancer (even in remission) (e.g., Díaz et al., 2008). Observational studies and clinical reports suggest that these same symptoms are frequently experienced by survivors of COVID-19 (Miaskowski et al., 2020; Vitale et al., 2020). Sleep disruption and fatigue can linger, become chronic, deplete motivation, create difficulties fulfilling activities of daily living, impede medical adherence, and limit social support and social connections (Tian et al., 2020; Yang et al., 2020).

What should the clinician advise the cancer/COVID-19 survivor who exhibits these symptoms? Available guidelines for treating fatigue and sleeplessness in cancer survivors are based on between-subject RCTs and do not provide evidence-based recommendations for a patient who also had COVID-19. No guidelines for treatment of these symptoms in COVID-19 survivors are yet available. Even if guidelines for patients with this combination of health conditions emerge, evidence found in between-subject RCTs will not predict how a specific patient will respond to the treatment. Instead, the clinician must resort to intuitions that reflect prior experience, the patient’s expectations and preferences, and careful consideration of alternative diagnoses and treatments (Spring et al., 2005). Based on intuition, a clinician may formulate a plan that is iterative and tailored to the patient. This intuitive plan, however, will lack a control condition and a formal assessment of the effectiveness of each iteratively tested treatment (Graber et al., 2012; Nendaz & Perrier, 2012). In contrast, the personalized experimental approach has the potential to identify the best course of treatment by making each person the subject of their own study. This design can offer individually tailored evidence to support decision-making processes for patients with multimorbidity who will never have a body of conventional RCT designs from which to draw.

## 4. What Treatment Should the Clinician Choose?

To begin with, the clinician must identify a treatment with the potential to improve the patients’ sleep and reduce fatigue. Besides its efficacy, the clinician must consider the treatment’s side-effects profile, cost, and effort required versus the resources available for the patient and clinician. Guidance based on anecdote, colleagues’ advice, and the empirical literature probably would indicate that bright light treatment and exogenous melatonin administration are treatments of choice for sleeplessness and fatigue (Bjorvatn & Pallessen, 2009; Morin et al., 2007) in general practice. Both symptoms represent, in part, disruption in circadian rhythm, which light therapy and melatonin are known to realign. However, a complex and idiosyncratic combination of circadian, pleiotropic, and disease-related factors may be responsible for the sleeplessness and fatigue in a specific patient. Given the challenges of identifying the specific mechanism in advance and then selecting the treatment, simply allowing a patient to test various promising treatments at the outset may be more helpful for finding the treatment that works for them. The main task for the patient and clinician is to find an effective treatment that alleviates a particular patient’s pressing symptoms. For patients with concurrent medical conditions, the challenges necessarily become more significant.

Suppose the clinician in our scenario decides to prescribe a course of melatonin, an over-the-counter pharmacologic. Melatonin is generally effective in reducing sleeplessness and fatigue (Rios et al., 2019; Wang et al., 2020), has a benign side-effects profile (Ferracioli-Oda et al., 2013), is low in cost, and is nonaddictive. The recommended dosage ranges from 0.3 milligrams (mg) to a maximum of 5 mg taken nightly (30–120 minutes before bedtime) (Morin et al., 2007).

One significant factor is that melatonin exhibits considerable HTE across patients; most people show improvement, but it varies across patients (e.g., Auld et al, 2017; Fatemeh et al., 2021). Recall that trial outcomes are commonly found to vary substantially across patients even when the population-averaged effect is statistically significant (Kent et al., 2018). In any case, no evidence currently exists about how a particular survivor of cancer and COVID-19 who also experiences sleeplessness and fatigue responds to melatonin. A personalized trial can provide the patient and clinician with information to make an informed and patient-centered decision about the right treatment for a specific individual.

## 5. Personalized Trial Development and Testing

Personalized trial designs resemble those for between-subject trials with four sequentially ordered stages, or phases, and each phase has a different purpose and answers different questions (DeMets et al., 2010; Umscheid et al., 2011). Upon successful completion of the first phase, the investigator moves to the next, and so on.

Conventional trials are designed to test whether patients assigned to the experimental treatment exhibit more benefits *on average* than the patients assigned to a control or placebo condition. Personalized trial design differs, however, because the personalized trial seeks the most effective treatment for a specific individual. The scenario for the survivor of cancer and COVID-19 will be used to illustrate the four developmental phases. Table 1 outlines the phases for personalized trials (righthand column). For purposes of comparison, the lefthand column describes phases for a conventional, between-subject trial. Finally, we should note that, to the best of our knowledge, this is the first attempt to formulate phases I–IV for personalized trials. Other scenarios may be viable.

### 5.1. Phase I Personalized Trial

Like between-subject phase I, the initial aim of a phase I personalized trial is to collect data about dosage and safety, but on an individual-by-individual basis. Thus, the primary outcomes concern maximally tolerable dosage and side effects. A range of doses would be tested from lowest to highest in each participant. The starting dose may be selected by extrapolating from preclinical (animal) studies or reliance on historical evidence about safe dosage. Then, by increasing the dosage incrementally or randomly within-subject and routinely monitoring side-effects, the maximally tolerated dose for each person can be determined. A surrogate marker would also be collected on a routine and a priori basis to detect whether that dosage is having an active response. For example, saliva, serving as a surrogate marker, can be analyzed to detect level of melatonin in the body in our clinical scenario. The data from phase I indicates the spread of the distributions of the maximally tolerated doses and the minimum active doses. Each patient’s recommended dose is defined as a dose or dose range that is tolerable and likely to be active (via a surrogate measure).

Typically, a small number of patients (e.g., 20) with the health conditions under study would each be recruited for their own phase I individual trial, conducted over several weeks. Unlike in a between-subject RCT, healthy volunteers would not be recruited since identifying the side effects and (surrogate marker) response of multimorbid patients is the aim of phase I. In our clinical scenario, each evening for 2 weeks, the participant would be instructed to take a melatonin pill at 6:00 p.m. Adherence would be monitored with smart (medication event monitoring system [MEMS] cap) pill bottles. The next morning, subjects would report via text message whether they experienced any side effects and rate their severity on a series of scales. On a regular basis, the participant would be asked to provide a saliva sample in specially provided vials to assess changes in the surrogate marker. Then, the volunteer would begin the next higher dose and follow the same procedures for two weeks and so on for up to 12 weeks. Typically, there would be two to three increments in doses (but, on occasion, more). The incremental sequence would be stopped at the point the individual reports significant side effects; their personalized, maximally tolerated dose is then determined jointly by the clinician and the patient.

After all participants have completed their personalized trials, the researchers will have data about the range of maximally tolerated doses, the range of active doses, and side effects. The researchers can then look at the distribution of the individualized data and decide if a single dose would be recommended or if a dose range (i.e., multiple doses) is more appropriate for phase II personalized trial design. In some cases, a trial oversight committee that includes nonclinicians (e.g., a statistician) may make these decisions.

### 5.2. Phase II Personalized Trial

This phase (often referred to as a therapeutic exploratory trial) would entail a larger series of personalized trials in individuals with the health condition under study. The main aims are to evaluate feasibility, continue to monitor for safety (i.e., side effects), and, most importantly, assess efficacy (i.e., symptoms, objective outcomes) of the intervention on a preliminary basis. Unlike phase II to prepare for a between-subject RCT, appropriate dosing, safety, and therapeutic efficacy are tested to identify what is appropriate for individual patients rather than the ‘average’ patient. Furthermore, a within-subject block sequence would be used in this phase rather than a between-subject design. Design strategies will differ depending on whether a single dose or a dose range emerged as tolerable and active in phase I. If the distribution of tolerated doses is very tight, each patient would receive the same (average) dose in their phase II personalized trial. For example, the trial might consist of a continuous series of 2-week blocks of the intervention (A) and a placebo-control (P), randomly assigned to patients in either a APPAAP or PAAPPA block sequence with regular measurement of symptoms and side effects.

Alternatively, the researcher might decide that a phase II personalized trial is not necessary if the distribution of dose needed for minimal efficacy is so tight as to indicate a universal response to the treatment.

Conducting a phase II personalized trial is more appropriate if the earlier phase I series revealed that the dose distribution was wide. Then, a two-step approach is advisable to determine the safe and effective dose for each patient first in a phase I personalized trial. Then, once an individualized dose is identified for each patient, each would be randomized in blocks to the individualized dose (A) and placebo (P) in the next step. Alternatively, the researcher might choose two or more tolerable doses (A and B) from phase I to test within-subject to find an optimal dose that is active. Patients would be randomly assigned to the ABBAAB or BAABBA block sequence. (A placebo-control block might also be added to the design, which would require a more complex block sequence.) Within-subject statistics can assess outcomes as a function of dose (block) looking for the most effective tolerated dose for that specific patient. It is worth noting that, if the ideal dose is very subjective and patient-based, phases I and II may be combined.

In our clinical scenario, the participants in phase II would exclusively consist of patients meeting inclusion criteria (in this case, survivors of cancer and COVID-19 who report severe sleeplessness and fatigue). Eligibility could be determined via an automated platform that delivers online surveys about demographic variables and health status and requests a series of ratings on questionnaires about recent symptoms. Patients who meet the inclusion criteria would then be enlisted by a study coordinator to participate in their own personalized trial. If a single dose is chosen for testing, each patient is involved in a single-person trial consisting of administration of the average melatonin dose (A) in blocks following the aforementioned single-dose strategy. If phase I fails to establish a single tolerated and active dosage, then two or more doses (e.g., A, B, C) may be tested. Then, patients will be randomly assigned to either an ABCCBA or CBAABC block sequence. For our scenario, six blocks of 2 weeks (i.e., 12 weeks in total) would likely be sufficient since melatonin is relatively fast-acting and has a short half-life. Independent of the specific design, side effects (via text messaging) and adherence (via smart, MEMS-cap pill bottles) would be monitored to evaluate feasibility as in phase I. Importantly, phase II would also assess preliminary treatment efficacy. For example, level of sleeplessness, fatigue, and related symptoms (e.g., affect, pain) may be queried each day or at the end of each week on validated and reliable self-report symptom questionnaires via automated text messages. In addition to subjective symptom reports, the clinician/researcher may assess objective indicators. For example, wearable remote sensors, such as activity monitors (e.g., Fitbit), could be used to monitor sleep time, activity, and heart rate to confirm subjective reports of sleeplessness and fatigue.

Personalized trials involve patients in receiving frequent messages and instructions and responding to inquiries over an extensive time period (of weeks or months). Patients’ experiences of acceptability and satisfaction with the protocol are important factors that affect the feasibility of trial implementation. This necessitates the collection of ratings and unstructured comments about acceptability and satisfaction (e.g., clarity of instructions, Fitbit usability, frequency of messaging) at the end of a block and at the conclusion of the trial. These factors may be an important consideration; even an efficacious treatment may not be feasible if patients report the treatment and personalized protocol to be unacceptable. Such information may lead clinicians/researchers not to proceed to phase III.

Typically, phase II trials involve a relatively small series of single-patient trials, thereby limiting the power to establish efficacy. However, these trials should yield sufficient evidence to indicate whether a larger-scale phase III trial is justified. More critically, for phase II personalized trials, each individual trial should have sufficient numbers of observations of outcome measures to attain adequate statistical power. The justification for moving to a phase III personalized trial is also different and will be discussed.

A residual or carryover effect of a prior block is a complicating factor. Washout phases (which are 1 or 2 weeks or more in duration) may be inserted between each dose phase (e.g., A, B, C) to eliminate any effect of the prior dose. Melatonin, however, has a relatively short half-life of 4 to 8 hours, making washout unnecessary (Arendt & Skene, 2005). Some treatments, of course, may have a long half-life. In such cases, an analytical washout approach could be used in longitudinal analyses, which include only the symptom scores from the second half of each block (when, presumably, any lingering effect of the prior block has disappeared).

To determine whether melatonin was superior to placebo control for reducing symptoms for individual patients, treatment effects can be assessed using an autoregressive model that includes the type of treatment as the main exposure, linearly adjusted for time (e.g., days since enrollment) as a covariate, and accounting for autocorrelations of the order 1 (Kronish et al., 2019). Statistical significance would be defined as *p* < 0.05. These individual statistical tests would indicate whether each patient does or does not exhibit a significant improvement in symptoms. Based on prior evidence, some patients would likely benefit from melatonin; some, however, would not, and others might even experience harm.

At the end of the 12 weeks, participants would receive visualizations of their personalized trial data in addition to survey responses about acceptability and satisfaction with the protocol to provide a basis for discussion about treatment preference with their clinician. The feedback would ultimately help the patient decide whether to continue taking melatonin at a suitable personalized dose level. Granting the somewhat arbitrary nature of the criterion, the researcher may select the dosage that ‘works’ for 75% or more of the participants. If, however, clusters of responders emerge—appreciable spread of dose response and activity—then the researcher may also choose to test multiple dosages in the next phase.

### 5.3. Phase III Personalized Trial

In this stage, the dosage(s) deemed to be safe and effective in phase II would be tested in a larger series of within-subject personalized trials to confirm efficacy and monitor side effects. (In a conventional RCT, patients would be assigned to one or more groups [treatments and controls/placebo].) Unlike phase II trials in which a control condition is not essential, phase III personalized trials frequently include placebo-control conditions as well as intervention components in the randomized sequence of interventions. Additional within-subject treatment arms may also be needed, as mentioned above, if the aim of the program of research is to conduct a comparative effectiveness contrast.

In phase III, the conduct of a larger series of trials permits further testing of personalized trial feasibility and an opportunity to determine, more accurately and precisely, the HTE that can be expected in a representative patient who undergoes a single personalized trial. A phase III trial focuses on therapeutic efficacy as the primary variable. However, a patient may decide that treatment side effects or acceptability are equally or more important to them. This consideration should be discussed thoroughly when, at the conclusion of the trial, the patient and clinician decide on the course of treatment. Another goal of the researcher is to conduct a sufficient number of phase III personalized trials to conduct a formal meta-analysis and subsequent meta-regressions (Zucker et al., 1997). The approach allows subgroups who responded similarly to be identified based on patient characteristics and contextual factors (Huber et al., 2007).

In the simplest case for our clinical scenario, each subject would receive melatonin, the active treatment (A), and a placebo-control condition (P). The placebo pills would have their own smart MEMS cap bottle. Each patient would be randomly assigned to either an APPAAP or PAAPPA block sequence. Our scenario consists of six blocks of 2 weeks (i.e., 12 weeks in total) in duration. (If more than one dosage is tested, then additional blocks B and C would be added to the design in a more complex counterbalanced sequence.) To reduce potential biases, when possible, neither the clinician nor the patient would know which of the treatments the patient is receiving (i.e., a double-blind study). Virtual and remote instructions and monitoring makes such blinding feasible.

**Figure 1.**
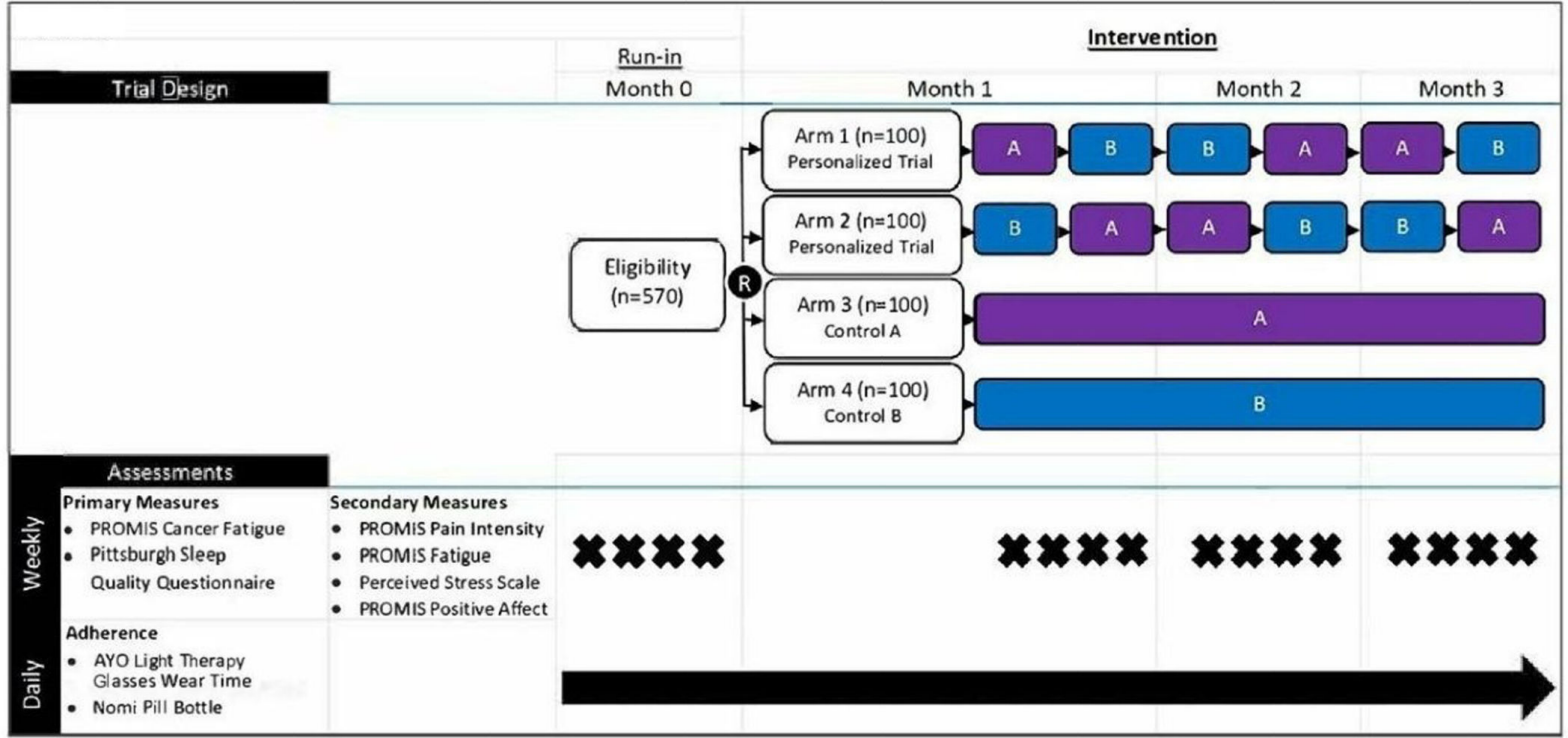
Design for comparing two treatments. Patient-Reported Outcomes Measurement Information System (PROMIS) is a series of reliable and valid self-report symptom assessments.

As in phase II, daily melatonin adherence would be monitored. Ratings of side effects would be queried remotely via text messaging, as would ratings of sleeplessness and fatigue on a daily or weekly basis. Ideally, remote sensors would record objectively verified sleep and activity. Figure 1 depicts a more complex personalized comparative effectiveness design (see Section 8.2) but lists the primary symptoms (i.e., fatigue and sleeplessness) and secondary symptoms (i.e., pain, perceived stress, positive affect) that also might be included for this simpler scenario. At the conclusion of the block sequence, within-subject comparisons of sleeplessness and fatigue ratings between blocks would be computed to test the efficacy of melatonin versus placebo control for each patient. Secondary outcomes, such as pain, positive affect, activity (steps per day) and sleep (hours per day) can be analyzed similarly. Statistical computations would follow those described for phase II: treatment effects can be assessed using an autoregressive model that includes the type of treatment as the main exposure, linearly adjusted for time (e.g., days since enrollment) as a covariate, and accounting for autocorrelations of the order 1. Individual statistical tests would indicate whether each patient does or does not exhibit a significant improvement in symptoms.

Critically, evaluations of the series of personalized trials conducted in the phase III design stage would indicate whether melatonin reduces sleeplessness and fatigue without side effects in different subgroups of participants. Bayesian analyses can be used to identify distinguishing features among those who did best on a specific intervention (Zucker et al., 1997). If such a characteristic is found, it can inform use of that intervention in the future.

As in phase II, participants receive personalized visual displays of their symptoms across baseline and treatment periods at the conclusion of the trial. This information informs the patient and their clinician about whether the treatment was beneficial; or if two or more treatments were tested, which had the most efficacy and which had the least. Reports about side effects, objective reports of activity and sleep, level of adherence, related symptoms, patient acceptability, and satisfaction with the protocol can also be integrated into decision-making between the patient and clinician about whether melatonin should be included in the patient’s regimen and thereby facilitate finely graded individualized care. The latter type of information is collected rarely or in-depth in between-subject RCTs, although how acceptable and satisfied the patient finds the protocol may influence decision-making more than therapeutic efficacy per se. Efficacy should be the primary outcome for phase III trials, but an individual patient may decide that effects on a secondary outcome (e.g., perceived stress, pain) are their main concern. In such cases, the patient may decide to continue (or not continue) with the treatment on that basis.

### 5.4. Phase IV Personalized Trial

If the treatment in phase III tends to improve sleep and fatigue without side effects—and heterogeneity of effects without clear subgroups indicates a similar response—moving to phase IV for personalized trials may be appropriate. This final phase may take the form of a personalized clinical trial service (offered to the public) that integrates an automated platform with all the other components for instruction, monitoring, and so on. The within-subject trial would permit the patient to identify the treatment that works best for them. In contrast, conventional phase IV trials are observational, and typically, only the treatment associated with significant (average) benefit (versus control/placebo groups in phase III) would be provided. As mentioned, personalized referral services were created at some institutions in the past, but they required labor-intensive, costly, manual operations and could not be maintained. Automated platforms that can integrate virtual, remote instruction, treatment, and monitoring are currently available, however. This means that survivors of both cancer and COVID-19 who also suffer from sleeplessness and fatigue could have the opportunity to enroll in their own personalized trial to determine whether they experience benefits from melatonin versus placebo control; if heterogeneity remains high, best science would prescribe that a test of usefulness needs to be conducted at the clinical encounter. Such a clinical trial service would be of benefit to patients with multimorbidity and the clinicians who treat them.

## 6. Use of a Run-In Period in Personalized Trials

Personalized trials, by necessity, require frequent and periodic monitoring to ascertain whether the patient experiences frequent and severe symptoms to qualify for enrollment. Patients must also be able to adhere to the trial protocol for extended periods; otherwise, data will be insufficient to provide meaningful feedback to the patient. Adding a ‘run-in’ period to the protocol can help to identify patients with frequent and severe symptoms and who are more apt to be adherent. For the COVID-19–cancer scenario, we may send eligible participants a kit with a MEMS pill bottle with placebo pills in it. During a four-week run-in, we ask that they take one pill each evening and report about symptoms of sleeplessness and fatigue on a weekly basis (in a survey sent by text message). Those whose symptom ratings fall below a criterion (based on prior normative data for the symptom questionnaire) or do not achieve at least 80% adherence of opening the pill bottle would be withdrawn from the study (e.g., > 24/30 days).

## 7. Are All Four Phases Necessary?

In some cases, all four design phases may not be necessary. Some interventions, particularly those involving medications, will probably have already undergone extensive testing to identify safe and efficacious doses associated with low HTE for particular health conditions and symptoms. In such cases, a clinician may immediately choose to conduct a phase III personalized trial with his/her patient. For patients with multimorbidity, however, immediate transition to a phase III trial may be unadvisable due to a scarcity of relevant phase I and II trials published in the literature. At the least, the clinician and patient should carefully deliberate about the feasibility of a phase III personalized trial for someone with multimorbidity with multiple treatment options.

## 8. Extension of Personalized Trials to Other Questions

Following successful conduct of the four phases, the clinician and patient have available a patient-centered approach to obtain personalized feedback about the degree to which a melatonin regimen provides useful benefits. Such feedback can help the patient and clinician make an informed decision about a future course of action. In our example, the patient may decide to continue to take melatonin regularly. Or, in the event the effects of melatonin are modest or even harmful, then the patient may be offered the opportunity to participate in a second personalized trial with a different treatment until a remedy that works for them is identified.

### 8.1. An Alternative to Melatonin

In our clinical scenario, a different treatment may need to be considered because melatonin is contraindicated for some persons (e.g., those taking corticosteroids). One alternative is light therapy, which is also hypothesized to realign circadian rhythm, improve sleep, and reduce fatigue (Tähkämö et al., 2019; van Mannen et al., 2016). In fact, bright-light therapy has been associated with improved sleep and reduced fatigue in medical populations that include cancer survivors (Johnson et al., 2018). Like melatonin, however, the effects of light therapy vary across patients; a personalized trial conducted with light therapy can determine whether the treatment is fitting for the individual patient. In this form of phototherapy, the patient is exposed to bright white light via a ‘light box,’ ‘a light visor,’ or special glasses.

Typically, a patient is instructed to use the device each morning for 30 minutes per day. In our clinical scenario, we recommend AYO light therapy glasses that use 470–475 nanometers (nm) light with an irradiance of 250 μW/cm2; this has been proven to be the most efficient wavelength for adjusting the internal body clock (Anderson et al., 2009; Tosini et al., 2016). AYO also can be fitted with Bluetooth technology so time-of-day and duration of use can be monitored remotely. AYO also can be set to emit dim-red light, a sham treatment, to serve as a placebo-control condition. The clinical trial phases described above can be modified by replacing melatonin with light therapy.

### 8.2. Comparing Different Treatments

Currently, little evidence to support clinicians in choosing between light therapy and/or melatonin exists; effects of both are likely to be variable due to heterogeneity in circadian, pleiotropic, and disease-related factors. A personalized comparative effectiveness trial can be designed to compare different treatments in the same patient. For example, to compare the effects of the two treatments, a patient could receive blocks of melatonin and light therapy in counterbalanced orders. As controls, placebo-comparators would also be administered simultaneously with the active treatment. For example, two treatment arms might consist of personalized (single-person, crossover) trials of two possible treatments: (A) melatonin (active treatment) plus dim-light (control) or (B) bright light therapy (active) plus placebo pill (control). The two arms would use an ABBAAB or BAABBA sequence, in which each block is 2 weeks (12 weeks in total). At the conclusion of their 12 weeks, Arm 1 and 2 participants would receive individualized feedback in a full report of their symptoms and decide whether bright light or melatonin best achieved symptom relief (see Figure 1 and Section 5.2). Discussion between the patient and clinician would weigh the benefits and liabilities (e.g., side effects, convenience) to determine whether to continue bright light or melatonin.

### 8.3. Testing Personalized Treatment Versus Group-Averaged Treatment

**Figure 2.**
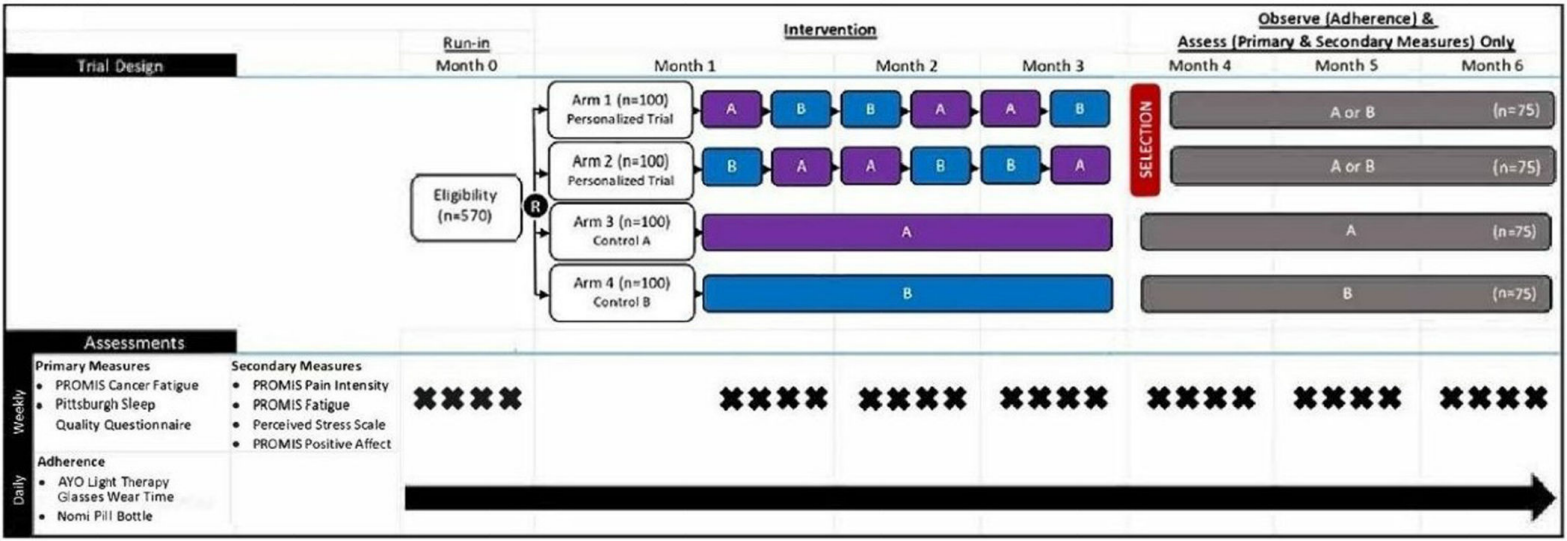
Design for hybrid, within- and between-subject randomized controlled trial (RCT). Patient-Reported Outcomes Measurement Information System (PROMIS) is a series of reliable and valid self-report symptom assessments.

Our working hypothesis has been that feedback from a single-person, crossover trial is more informative and more beneficial to a patient than a group-averaged treatment. This hypothesis can be tested in a four-treatment-arm hybrid design that embeds personalized trials in a between-subjects design (see Epstein & Dallery, 2022). Figure 2 depicts this hybrid design. The single-person, crossover trials—Arms 1 and 2—are identical to those described above. Arms 3 and 4 are standardized, 12-week control ‘Single Treatment’ arms of only melatonin or bright-light therapy to which participants are randomly assigned. At the conclusion of their 12 weeks, participants in Arms 1 and 2 receive feedback in a full report of their symptoms and decide whether to continue bright light or melatonin for the next 12 weeks (see Section 5.2 about data visualizations provided to the patient). In the control arms, randomized treatment is continued and participants are assessed for another 12 weeks (during the ‘Observe and Assess’ period). Monitoring of symptoms and treatment adherence continue during the second 12-week period (see Figure 2).

If we note improvement in averaged monthly fatigue and sleep between the Personalized arms (1 and 2) versus Single Treatment arms (3 and 4) from Run-In Month and Months 4–6, we could conclude that personalized feedback has greater clinical efficacy than averaged feedback from between-subject trials. Similarly, if averaged monthly treatment adherence improves between Personalized arms (1 and 2) versus Single Treatment arms (3 and 4) from Run-In Month to Month 6, then patients are more adherent to a treatment when they receive personalized feedback about its comparative effectiveness. Our working assumption is that personalized, comparative feedback will lead to informed selection and better adherence, but some patients may opt for a less effective treatment because they find adherence to the alternative to be easier and convenient. Identifying health conditions and treatments that are best suited for personalized trials offers the opportunity to learn whether an idiothetic or nomothetic treatment plan is more informative and beneficial.

## 9. Personalized Trials Can Address Special Problems of Multimorbidity

The personalized trial paradigm is applicable to a wide range of patients with different patterns of multimorbidity. Substance use disorders (e.g., alcoholism, opiate addiction) commonly co-occur with anxiety disorders. In such cases, personalized trials comparing different medications or (reversible) behavioral interventions may be appropriate as the effects of both medications and behavioral interventions are associated with high HTEs. A patient who has diabetes and hypertension may be tested in a personalized trial comparing exercise and medications to determine which works best (Shaffer et al., 2018). Personalized trials, however, are not limited to concordant comorbid conditions that share care goals and risk management, such as diabetes and hypertension (Piette & Kerr, 2006). Discordant conditions—not directly related in either their pathogenesis or management and without an underlying predisposing factor (e.g., diabetes and asthma)—also are amenable to single, within-subject crossover trials.

Personalized trials also can serve patients with rare conditions or with an infrequent combination of rare conditions who are often not tested in studies of interventions because too few participants with similar characteristics can be found for a between-subject RCT. The costs associated with testing and drug development are enormous and currently depend in the main on between-subject designs. The likelihood that patients with a combination of health conditions have been systematically tested in RCTs is unlikely. Nonetheless, prescription and over-the-counter drugs often can serve as primary sources of relief to patients with multimorbidity. The personalized trial offers a more efficient and less costly option to determine what is both safe and effective on an individual basis.

The various health and everyday life challenges faced by patients with concurrent health conditions can create numerous personal impediments. The remote/virtual technologies integrating care in personalized trials have the potential to reduce in-person clinic time and cost/travel burden and thereby facilitate patient access to care. Remote sensors and daily subjective reports that are integrated with personalized protocols can provide clinicians with information about the patient’s health trajectory that is currently unavailable in the conventional between-subject RCT protocol. We acknowledge, however, that much still needs to be learned about health conditions and areas of functioning for which these new approaches may not provide benefits.

## 10. Making Personalized Trials Feasible and Acceptable to Patients

Persons who experience co-occurring conditions and their sequelae must contend with medical issues and demands of everyday life, which are currently further exacerbated by the COVID-19 pandemic. Such persons may be unreceptive to personalized trials that require them to respond to text messages for instructions, respond to daily and weekly survey questions, and/or wear remote sensors for an extended period. Alternatively, patients dealing with multimorbidity may be more willing to participate in personalized trials than in between-group RCTs *if* they believe the results of the personalized trial will directly benefit *their* care, which is traditionally not a goal of between-groups research. Two studies examined receptivity to personalized trials in a nationally representative sample of participants with two or more chronic conditions. After they were provided with different personalized trial scenarios, Derby et al. (2021) found that 82% of those sampled were interested in participating in personalized trials. A large proportion (nearly 90%) reported finding it very appealing that participation could yield information about whether one treatment is more effective than another for individual patients (Moise et al., 2018). Design features such as blinding, treatment type, and overall trial duration did not significantly influence their preferences. Personalized trial prototypes that minimized out-of-pocket costs and only required brief daily instructions and subjective reports was a decided preference (Moise et al., 2018). Personalized trial designers and public health officials should consider ways to limit out-of-pocket costs associated with personalized trials and consider facilitating brief self-monitoring. For our clinical scenario, the cost of melatonin (3 mg) is less than $10 for 120 capsules; light-therapy glasses cost about $150. In general, text messages require 2 to 3 minutes to read and follow. In summary, persons with multiple chronic conditions do not report any aversions and appear to be willing to participate in personalized trials; in fact, they see some benefits in being able, themselves, to test which treatment is most effective for them. This kind of direct feedback enhances clinician-patient shared decision-making in personalized treatment decisions, which is not possible in a between-subject RCT.

## 11. Limitations

Personalized trials will not be an option for all patients with multimorbidity. For some, health conditions may require treatments that are irreversible (i.e., they cannot return to baseline) or cannot be withdrawn. A no-intervention baseline block is commonly used as an initial condition in many personalized studies, but this would be contraindicated for patients who are very ill. Epstein and Dallery (2022) also suggest that the intervention should have a relatively immediate effect on the outcome. If the observation of effects takes weeks to months for a given intervention, a personalized design may not be optimal unless the investigator is willing to plan a lengthy study. This may be discouraged due to the need to frequently monitor the patient. Furthermore, some patients may be hesitant to enroll in a trial requiring repeated measurements. Illnesses that are severe and progressive may be poor candidates for personalized trials because a stable baseline cannot be obtained. Conducting personalized trials may be challenging, and they are not a panacea for all clinical conditions and patients. Determining an appropriate randomization scheme, outcome measurement, and analysis requires methodological and statistical knowledge. Allowing for an adequate number of crossovers is necessary, especially if the patient’s condition is not stable. In such cases, a personalized trial may be counter-indicated. Providing blinded treatments may involve coordination with a hospital pharmacy or a commercial operation. And, of course, personalized trials are not appropriate for all situations. Furthermore, treatments with an exceedingly long half-life that do not lend themselves to washouts and treatments that are unethical to withdraw or reverse are also inappropriate. Finally, unblinded personalized trials should be interpreted with caution given the bias that may be introduced by failure to blind (Gabler et al., 2011).

Although personalized trials designed for certain categories of primary symptoms may be counter-indicated, secondary symptoms, such as fatigue and quality of life, may be appropriate as targets for personalized behavioral interventions. If these interventions ameliorate such secondary symptoms, they can potentially improve coping and adherence to ongoing or future medical procedures or treatment regimens.

## 12. Conclusions

Our aim has been to illustrate how personalized trials incorporating recent advances in computing and virtual/remote technologies offer an approach to revolutionize the way we identify the best treatments for those with multimorbidity. In addition, embedding a series of personalized trials in a hybrid, within- and between-subject RCT design can advance knowledge about whether personalized trials are preferred and more effective than between-subject RCTs, and are suitable for broad use in research and practice. Finally, patients involved in personalized trials, unlike the conventional RCT, can draw immediate benefit from the trial in that a determination of which intervention is likely to benefit them can be made.

## Figures and Tables

**Table 1.** Comparison of between-subject and personalized (N-of-1) trial phases.

Conventional (Between-Subject) Clinical Phases	Personalized (N-of-1) Clinical Phases
**Phase I**: Identify the average tolerated and safe dosage for a new drug or intervention in a sample. Also measure a surrogate marker to signal an active response to the treatment.	**Phase I**: Identify tolerated and safe dosage on an individual-by-individual basis. Also measure a surrogate marker to signal an active response to the treatment.
Participants: A small group of healthy volunteers (e.g., 20 persons) and a smaller number of patients with the medical condition under study.	Participants: A small number of patients (e.g., 20 persons) with the medical condition under study.
Procedure: The first few participants receive a very low dose of the treatment. The next few participants get a higher dose in the event the only experience minor side effects. A surrogate marker (e.g., level of medication in blood) also is periodically assessed. This process continues until the maximally tolerated dose is reached (and the surrogate marker indicates a change). A safe and tolerated dose at which a surrogate marker also registers a response is established by averaging values across participants.	Procedure: Conduct dose-titration on an individual patient basis. A range of doses is tested from lowest to highest in each participant while side effects and changes in surrogate marker are monitored at each dosage level. Data indicate the spread of the distribution of the maximally tolerated dose or minimum effective dose. Each patient’s recommended dose is defined as a dose or dose range that is tolerable and likely to be active (via a surrogate measure).
**Phase II**: Test whether the dose/range of doses found safe in phase I improves symptoms on a preliminary basis and continues to be safe and well-tolerated in a larger sample of persons with the health condition under study.	**Phase II**: Test whether a single dose/range of doses showing safety and response in phase I continue to be safe and well-tolerated and show preliminary evidence for symptom improvement on an individual basis for patients with the health condition.
Participants: A larger group of patients with the health condition.	Participants: A larger sample of patients each assigned to their own personal, within-subject trial.
Procedure: Everyone enrolled may get the same dose that was established in the earlier phase, although, in some cases, patients may be randomly assigned to groups to receive different doses to see which provides the best balance of safety and response. Treatment outcome efficacy is assessed by comparing symptoms at baseline versus during and/or following treatment. If different doses are randomly assigned to subsets of patients, then the average differences in outcomes between dosage groups would be statistically tested. Placebos (i.e., inactive treatments) are not typically used in between-subject phase II trials because phase II trials involve relatively small samples (i.e., the data are unlikely to yield statistically significant effects), though they may be used to compute effect size to inform sample size for the next phase.	Procedure: 1. If the dose distribution in phase I is very tight then a single dose could be tested in all patients’ personalized trials. 2. If the distribution is wide, a two-step approach is advised: first, conduct a personalized trial for each patient and, once an individualized dose is identified for each patient, each would be randomized in blocks to the individualized dose (A) and placebo (P). Alternatively, the researcher might choose two or more tolerable doses (A and B) from phase I to test within-subject to find an optimal dose that is active and without side effects. Patients would be randomly assigned to ABBAAB or BAABBA block sequence. (A placebo-control block might also be added to the design, which then would require a more complex block sequence.) Within-subject statistics would evaluate outcomes as a function of dose (block) for each participant. If therapeutic benefit is evident for some patients, more single-participant trials will be conducted in the next phase.
**Phase III**: To confirm efficacy and monitor side effects of the new drug or treatment in a larger sample of patients.	**Phase III**: To further test efficacy, feasibility, and heterogeneity of treatment effects (HTE) that can be expected in a representative sample who underwent a single personalized trial.
Participants: This step usually involves several hundred or more patients with the health condition in order to ensure the trial has sufficient statistical power to detect a statistically significant effect and any rare, non-benign side effects.	Participants: A larger sample of several hundred patients with the health condition; each participant is assigned to their own within-subject crossover trial. The larger series of trials provides sufficient data to conduct a meta-analysis and meta-regression of these trials.
Procedure: Patients are randomly assigned to the new drug/intervention versus placebo or control condition. Symptoms, adherence, and side effects are measured periodically at baseline, mid-study, and conclusion but not as frequently as in a personalized trial. Statistical testing compares the average improvement of the patients assigned to the new treatment condition versus the placebo/control.	Procedure: In the simplest case, each subject would receive active treatment (A) and a placebo-control condition (P) but randomly assigned to either an APPAAP or PAAPPA block sequence. (If more than one dosage is tested, then additional blocks—B and C—would be added.) At the conclusion of the block sequence, within-subject comparisons of symptoms and safety between blocks would be computed to test the efficacy of treatment versus placebo/control for each patient. In addition, meta-analysis and meta-regression would be conducted to identify distinguishing features among those who did best/worst on a specific intervention.
**Phase IV**: The treatment is made available to the public. Researchers track its safety in the general population, seeking more information about benefits and optimal use. These types of questions may take many years to answer and may involve thousands of people.	**Phase IV**: If the treatment in phase III significantly improved sleep and fatigue without side effects, and heterogeneity of treatment response was quantified in a large, representative sample without clear subgroups who showed a similar response, moving to phase IV may be appropriate. Clinical personalized trial services can be offered remotely to the public. Automated platforms integrate virtual, remote instruction; treatment and monitoring on an individual basis; and personalized feedback.
